# Supplementary material for: MetaRibo-Seq measures translation in microbiomes
Source: Nat Commun. 2020 Jun 29;11:3268. doi: 10.1038/s41467-020-17081-z (PMC7324362; doi:10.1038/s41467-020-17081-z)
Supplement: Supplementary file 10 — Supplementary Data 7 [file 41467_2020_17081_MOESM10_ESM.zip › File2/Confidence_High_Taxonomy/179091_out.krona.html]

Javascript must be enabled to view this page.

members
magnitude
magnitudeUnassigned
count
unassigned
taxon
rank

179091\_out

14

superkingdom
2
14

phylum
14
976

200643
14
class

171549
14
order

171552
14
family

genus
14
838


SRS012969\_contig\_number\_10910SRS023526\_contig\_number\_37069SRS077849\_contig\_number\_21693
species
3
2292365

species

SRS017849\_contig\_number\_contig-100\_8708.8708
1947118
1

species

SRS013687\_contig\_number\_contig-100\_5939.169490SRS015782\_contig\_number\_contig-100\_2263.236770SRS023914\_contig\_number\_contig-100\_4724.142720SRS044535\_contig\_number\_contig-100\_394.44784SRS047044\_contig\_number\_contig-100\_5647.86370SRS047433\_contig\_number\_11097SRS049896\_contig\_number\_11720SRS077641\_contig\_number\_20221SRS1041116\_contig\_number\_4006SRS146764\_contig\_number\_27785
10
165179
